# Efficacy of extended release formulations of Natular™ (spinosad) against larvae and adults of *Anopheles* mosquitoes in western Kenya

**DOI:** 10.1186/s12936-020-03507-y

**Published:** 2020-11-26

**Authors:** John E. Gimnig, Maurice Ombok, Nabie Bayoh, Derrick Mathias, Eric Ochomo, William Jany, Edward D. Walker

**Affiliations:** 1 Centers for Disease Control and Prevention, Division of Parasitic Diseases and Malaria, Atlanta, GA USA; 2 Kenya Medical Research Institute, Centre for Global Health Research, Kisumu, Kenya; 3 Present Address: PMI VectorLink Project, Abt Associates, Lusaka, Zambia; 4 Present Address: Florida Medical Entomological Laboratory, University of Florida, Vero Beach, FL USA; 5 Clarke International, St. Charles, IL USA; 6 Department of Microbiology and Molecular Genetics, Michigan State University, East Lansing, MI USA

**Keywords:** Mosquitoes, Malaria, Larval source management, Spinosad

## Abstract

**Background:**

Larval source management is recommended as a supplementary vector control measure for the prevention of malaria. Among the concerns related to larviciding is the feasibility of implementation in tropical areas with large numbers of habitats and the need for frequent application. Formulated products of spinosad that are designed to be effective for several weeks may mitigate some of these concerns.

**Methods:**

In a semi-field study, three formulations of spinosad (emulsifiable concentrate, extended release granules and tablet formulations) were tested in naturalistic habitats in comparison to an untreated control. Cohorts of third instar *Anopheles gambiae* (Diptera: Culicidae) were introduced into the habitats in screened cages every week up to four weeks after application and monitored for survivorship over three days. A small-scale field trial was then conducted in two villages. Two of the spinosad formulations were applied in one village over the course of 18 months. Immature mosquito populations were monitored with standard dippers in sentinel sites and adult populations were monitored by pyrethrum spray catches.

**Results:**

In the semi-field study, the efficacy of the emulsifiable concentrate of spinosad waned 1 week after treatment. Mortality in habitats treated with the extended release granular formulation of spinosad was initially high but declined gradually over 4 weeks while mortality in habitats treated with the dispersable tablet formulation was low immediately after treatment but rose to 100% through four weeks. In the field study, immature and adult *Anopheles* mosquito populations were significantly lower in the intervention village compared to the control village during the larviciding period. Numbers of collected mosquitoes were lower in the intervention village compared to the control village during the post-intervention period but the difference was not statistically significant.

**Conclusions:**

The extended release granular formulation and the dispersible tablet formulations of spinosad are effective against larval *Anopheles* mosquitoes for up to four weeks and may be an effective tool as part of larval source management programmes for reducing adult mosquito density and malaria transmission.

## Background

Malaria control efforts since 2000 have resulted in dramatic reductions in morbidity and mortality. Between 2000 and 2015, infection prevalence was halved, and the incidence of clinical malaria fell by 40%. Models suggested that much of the decline was due to the scale-up of insecticide-treated nets (ITNs) and indoor residual spraying (IRS), which together were credited with over 80% of the reduction in malaria prevalence [1]. However, in recent years, the pace of the decline has slowed and in some countries, the incidence of malaria has increased [2]. Reasons for the stagnating progress in malaria control are varied but likely include plateaued funding [2] as well as the rise and spread of insecticide resistance [3]. Another potential factor contributing to the stabilization of malaria is the increasing importance of outdoor transmission as ITNs and IRS primarily target indoor feeding and resting mosquitoes [4].

While ITNs and IRS are the core interventions recommended for malaria vector control, larval source management is recommended as a supplemental intervention where feasible [5]. Larval source management includes habitat modification, habitat manipulation, larviciding and biological control such as the introduction of larvivorous fish. Cluster randomized controlled trials have demonstrated the efficacy of ITNs in reducing malaria burden [6] and while high quality epidemiological trials of IRS are lacking, its effectiveness has been documented through a long history of programmatic implementation [7]. A systematic review of larval source management reported reductions in malaria incidence and prevalence in several studies in east Africa, and south and southeast Asia, while a trial in west Africa in an area with extensive flood plains showed no impact on either prevalence or incidence [8]. A subsequent systematic review focusing on larviciding found that it probably reduces incidence and may reduce prevalence in areas with aquatic habitats less than 1 km^2^ but the strength of the evidence was considered low to moderate. The same review could not draw a conclusion on whether larviciding affected incidence or prevalence in areas with aquatic habitats greater than 1 km^2^ [9]. Based on these data and concerns about its operational feasibility, the World Health Organization (WHO) recommends larval source management only for areas where high coverage of ITNs or IRS has been achieved and where the habitats are “few, fixed, and findable” [5].

Despite the limited recommendation, there is increasing interest in larviciding for a variety of operational situations, including use as a complementary intervention to ITNs and/or IRS where insecticide resistance is spreading and/or intensifying, as a tool to address outdoor biting by mosquitoes that may avoid insecticides applied to nets or walls, and in areas approaching elimination where time-limited implementation of larviciding may aid in the final push to elimination. To date, most larviciding applications in sub-Saharan Africa have been implemented with *Bacillus thuringiensis* var. *israelensis* (*Bti*) or *Bacillus sphaericus* (*Bs*) [10]. These are effective larvicides with no known adverse environmental effects and despite widespread use in developed countries, their complex mode of action has limited the development of resistance [11–13]. However, the formulated products used to date have limited durations of efficacy [14]. In sub-Saharan Africa, where development of *Anopheles gambiae* from egg to adult may occur within a week [15], frequent application of larvicide formulations with less durable effect is needed, limiting the feasibility of larviciding and increasing the likelihood that habitats will be overlooked.

Alternative insecticides or insecticide formulations with extended durations of activity may increase the feasibility of larviciding and expand the range of areas that may be targeted. One insecticide that has been formulated for longer residual efficacy is spinosad, which is composed of spinosyns, a family of chemicals found in the bacteria, *Saccharopolyspora spinosa*. There are over 20 natural and 200 synthetic spinosyns. Spinosad is comprised of spinosyn A and spinosyn D and has been formulated as a broad-spectrum insecticide that is active against mosquitoes through both contact and ingestion [16, 17]. Spinosad allosterically affects the nicotinic acetylcholine receptor causing hyperexcitation of the nervous system. It does not show cross resistance with insecticides currently used in public health and is generally considered safe to humans and to the environment. The initial recommendation for spinosad products from the WHO Pesticide Evaluation Scheme indicated that spinosad is practically non-toxic to birds, moderately toxic to fish and highly toxic to honey bees [18]. For mosquito control, it is available under the brand name Natular™ (Clarke Mosquito Control, St. Charles, IL, USA) in multiple formulations including an extended release granular form and a dispersible tablet form that are designed to provide continuous release for up to 30 days [18, 19].

To assess the potential of spinosad for malaria vector control in sub-Saharan Africa, multiple formulations of Natular were tested in semi-field, artificial habitats and then a pilot of the efficacy of larviciding was conducting using two of the longer lasting Natular formulations in a field setting in western Kenya.

## Methods

### Semi-field evaluation

**Fig. 1:**
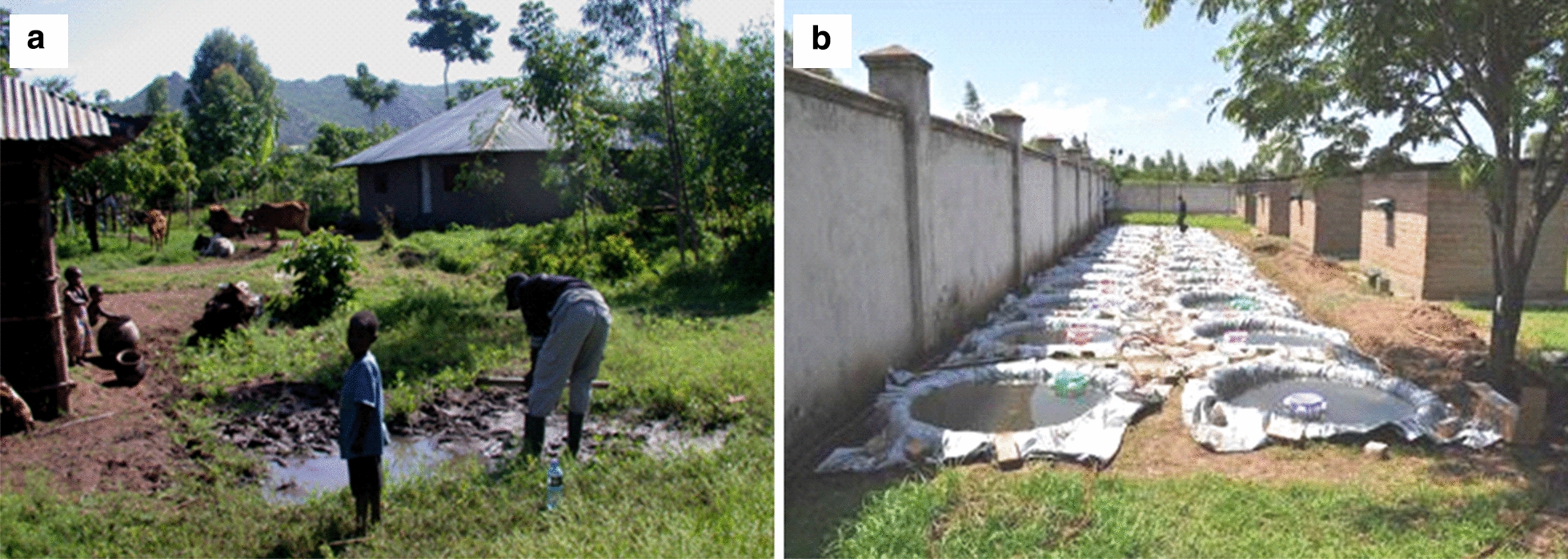
**a** A typical larval habitat for *Anopheles gambiae s.l*. in a village near the KEMRI-Centre for Global Health Research, Kisian, Kenya; **b** array of pits along the security wall of the KEMRI-Centre for Global Health Research campus

The semi-field evaluation was conducted on the grounds of the Centre for Global Health Research of the Kenya Medical Research Institute, located in Kisian village, approximately 10 km west of the city of Kisumu, Kenya from May 29 to June 30, 2009. Natural larval habitats of the primary vectors of malaria in western Kenya are typically small ground water habitats resembling mud puddles (Fig. 1a). Naturalistic habitats (Fig. 1b) were created by digging pits in the ground, each 1.52 m in diameter and 0.36 m in depth. To prevent water from leaching into the ground, each habitat was lined with polyethylene sheets. Soil was then added on top of the sheets to a thickness of 2 cm. Water was added to the pits to a depth of 35 cm. The habitats were stable with minimal loss of water to evaporation and no water was added during the course of the experiment. There was occasional rainfall of a non-inundative nature and the gap left between the predetermined level of water and the top edge of each habitat was enough to accommodate any rain that occurred during the course of the experiments without overflowing. Water temperature was measured daily in a subset of habitats during the entire experimental period using a mercury thermometer. The temperature ranged from 23 to 33 °C, with an average of 26.7 °C.

The following treatments were applied to habitats in a randomized experimental design: (1) Natular tablet formulation (T30 8.33% w/w; application rate: 1 tablet/habitat); (2) Natular extended release granular formulation (XRG 2.5% w/w; application rate: 11.2 kg/ha or 1.12 g/m^2^); (3) Natular emulsifiable concentrate formulation (EC 2 lb; application rate: 0.021 ml/m^2^); and (4) untreated control. The labelled rates are 1 tablet per 9.29 m^2^ habitat up to a depth of 60 cm for the Natular T30, 5.6–22.4 kg/ha for the Natular XRG and 0.020 ml/m^2^ for the Natular EC with higher rates applied to habitats with more organic material. A total of 20 habitats were used in the experiments with 5 replications for each treatment.

Thirty 3rd instar larvae of *An. gambiae *sensu stricto (*s.s*.), Kisumu strain, were placed in bioassay cages that floated at the surface of the water in each habitat. The bioassay cages were constructed from plastic baskets that were 23 cm in diameter and 15 cm deep. The baskets were lined with netting material with a mesh size of 3 holes per mm and the same netting material was used to cover the bioassay cages to prevent entry of other mosquitoes or predators. Styrofoam was affixed to the sides of the baskets to allow them to float. Larvicide was applied on day 0 and then five different cohorts of larvae were introduced at approximately weekly intervals from week 0 to 4 corresponding to days 0, 9, 16, 23 and 31. However, due to low mortality and limited numbers of mosquitoes, the Natular EC formulation was only evaluated at weeks 0 and 1. The number of larvae remaining was recorded at 24, 48, and 72 h after seeding larvae into the floating cages for each cohort. Mortality was calculated as the number added to the cage at the beginning of each cohort (30) minus the number of larvae remaining in the floating cages. Percent mortality was then calculated by dividing by 30. After each cohort was assessed at 72 h, cages were removed, and immature mosquitoes that remained alive were held in the laboratory in water collected from the semi-field habitats up to 9 days after the initial introduction into the cages to allow for pupation. Bioassay cages were then cleaned with soap and water and then rinsed with clean water. The cages were labelled according to the habitats they were originally placed in and were returned to the same habitats to further avoid contamination at the beginning of the next week with new cohorts of larvae.

It was observed that wild mosquitoes were developing in the semi-field habitats. Therefore, on days 11 through 16 after application of larvicide, the habitats were inspected for 4^th^ instar larvae and pupae which were removed from the habitats and held in the laboratory until adult emergence.

### Field evaluation

**Fig. 2:**
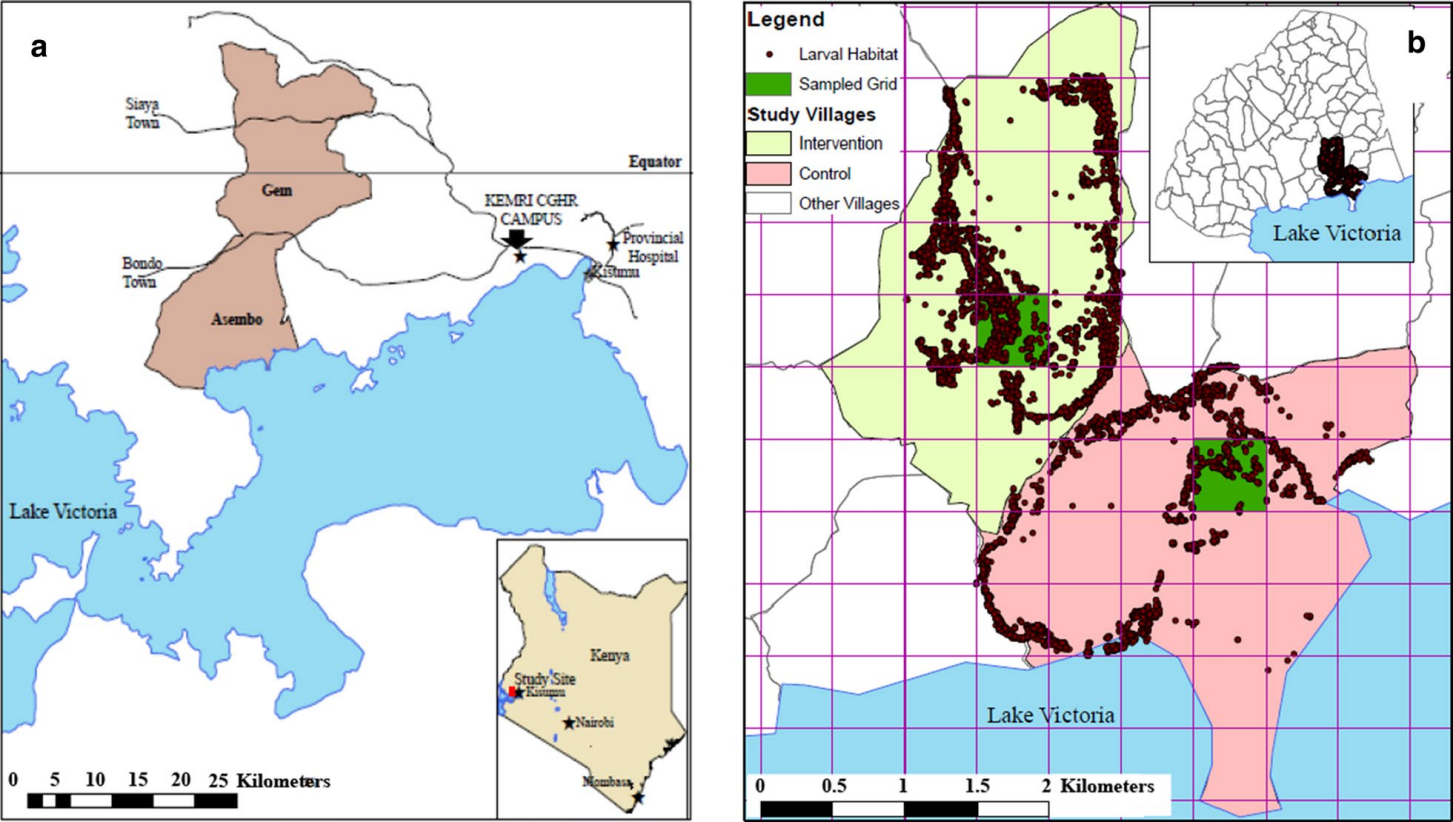
Overview of experimental designs: **a** Location of the KEMRI Centre for Global Health Research (CGHR), Kisian western Kenya where the semi-field studies were conducted and Asembo where the field trials were conducted; **b** location of the intervention and non-intervention villages in Asembo. Shaded areas indicate the 500 × 500 m where larval surveillance was conducted

Based on the results from the semi-field, the field trial was designed and implemented between March 2011 and September 2012, to test the efficacy of larviciding in reducing the density of adult and larval mosquitoes in two villages in western Kenya. The villages were in the Asembo Bay area, located along the shores of Lake Victoria, 50 km west of Kisumu city. The area is part of a Health and Demographic Surveillance System (HDSS) managed by the Kenya Medical Research Institute and the US Centers for Disease Control and Prevention KEMRI-CDC [20] (Fig. 2a). Residents of Asembo Bay experience persistent malaria transmission despite high coverage of ITNs. The study area is characterized by rolling hills bisected by permanent or semi-permanent streams that are flooded during the rainy seasons, which in turn floods low-lying parts of the two study villages. Average annual rainfall is approximately 1200 mm with maximum precipitation occurring during the long rains from March to May and a less intense amount of rainfall during the short rainy season in November–December. Average daily temperatures range from 19 to 29 °C.

Most residents of the area are of the Luo ethnic group. Houses are grouped into compounds of related family members and are separated from each other by farmland. Most of the inhabitants are subsistence farmers who cultivate maize, sorghum, millet, and vegetables. Livestock including cattle, sheep and goats are kept by many of the residents. The locations of the villages are presented in Fig. 2b.

Malaria transmission in this area has historically been very high with entomological inoculation rates once estimated to be over 300 infectious bites per person per year [21]. *Plasmodium falciparum* is the primary malaria species and the primary vectors are *An. gambiae* s.s.*, Anopheles funestus* and *Anopheles arabiensis*. The area has a history of net use starting with an intervention trial beginning in the late 1990s [22, 23] and subsequent scale up throughout western Kenya through mass-campaigns [24] and routine distribution to pregnant women and infants [25]. As a result, transmission has been substantially reduced and the *Anopheles* species composition observed in houses shifted from predominantly *An. gambiae s.s.* and *An. funestus* to predominantly *An. arabiensis*, although more recent studies indicate a resurgence of *An. funestus* associated with resistance to pyrethroid insecticides [26].

### Larvicide treatment

Beginning in March 2011 and ending in September 2012, all larval habitats in the intervention village were treated with the Natular XRG granular formulation in the small, shallow habitats at an application rate of 11.2 kg/ha and the Natular T30 tablet formulation in larger, deeper habitats on a 3-week cycle. Regular entomological assessments were done in both the intervention and control villages. All larval habitats were geo-referenced and the data analysed using a GIS system (ArcGIS version 9.3, ESRI, Redlands, CA) to assist in tracing back the habitat. Locations of the larval habitats identified and mapped in each village are shown in Fig. 2b.

### Larval sampling

Stage specific presence or absence and density of the immature stages of *Anopheles* mosquitoes were determined once every two weeks in all habitats within a 500 × 500 m grid in each village (Fig. 2b) by trained larval monitors not involved in larvicide application. To measure larval density and pupal production, each habitat was inspected by eye to determine presence or absence of larvae. Within each metre along the habitat edge, the area with the highest concentration of larvae was sampled using a standard 300 ml dipper. If no larvae were seen, any area within the metre was sampled. Larvae were categorized and counted by instar.

In addition, 30 sentinel habitats were randomly selected after an initial mapping of all habitats within the village. The sentinel habitats were relatively stable habitats that were monitored every five days. Assessment of habitat occupancy and larval density were measured as described above.

### Adult sampling

Indoor resting densities were estimated using pyrethrum spray collections (PSC) every two weeks from March 2011 at the start of the intervention to December 2013, which was 15 months after the end of the intervention period. Each month, one compound was randomly selected in the intervention and non-intervention villages as a reference point and PSCs were carried out in the nearest 20 houses. PSCs were performed by laying white sheets upon the floor and over the furniture in all rooms within each house. The house was then sprayed with 0.025% pyrethrum emulsifiable concentrate with 0.1% piperonyl butoxide mixed into 5 L of kerosene with one collector who sprayed outside around the eaves and a second collector who sprayed the roof and walls inside the house. The house was then closed for 10–15 min after which knocked down mosquitoes were collected from the sheets and transferred to the laboratory on moist filter paper inside petri dishes. Mosquitoes were identified using standard morphological keys [27].

### Statistical analysis

The semi-field data were entered into a Microsoft Excel workbook (Microsoft Corporation, Redmond, WA, USA) and imported into SAS 9.4 (SAS Institute, Cary, NC, USA) for statistical analysis. Mosquito mortality after 24, 48 and 72 h was compared using logistic regression using PROC GENMOD. Predictor variables included treatment, week as an ordinal categorical variable and an interaction between treatment and week. Models were adjusted for repeated measures on the same artificial habitats over time assuming an autoregressive correlation structure.

The field evaluation data were collected using personal digital assistants (PDA) for both adult and larval collections. Data were downloaded and managed in Microsoft Access workbook (Microsoft Corporation, Redmond, WA, USA) or Microsoft Excel then imported into SAS 9.4 for statistical analysis. Habitat occupancy over time was expressed as the proportion of habitats with larvae or pupae while larval density was expressed in terms of the number per habitat, the density per 1 m^2^ area of habitat sampled or the number per dip. Separate models with the number of immatures per habitat as the outcome were run for early (1st and 2nd instar) and late (3rd, 4th and pupa) pre-adult stages as well as for the total number of immatures. Statistical comparisons were done using negative binomial regression with the number of larvae per dip as the outcome variable and treatment as the only predictor variable.

Adult densities for each village and period of the study were calculated by dividing the total number of adult mosquitoes collected by the number of houses sampled. The study period was divided into the intervention period (March 2011 to September 2012) and the post-intervention period (October 2012 to December 2013). Adult numbers were modelled using negative binomial regression. Predictor variables included village, period and an interaction term. Reported net use and the presence of open or closed eaves were included as covariates. The models were adjusted for clustering at the household level assuming an autoregressive correlation structure. Adult numbers were analysed by species [*An. gambiae *sensu lato (*s.l*.) and *An. funestus*] and by total *Anopheles*.

## Results

### Semi-field evaluation

**Fig. 3:**
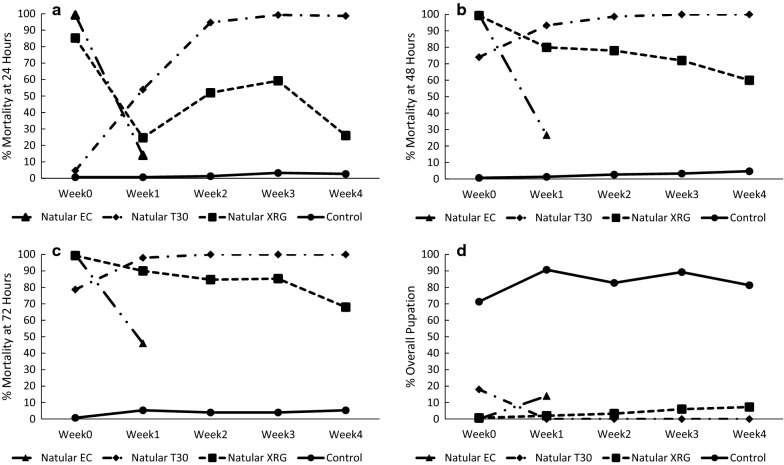
Mortality and pupation of cohorts of 3rd instar *An. gambiae* Kisumu strain after introduction into semi-field habitats for up to 4 weeks after treatment with different larvicide formulations. **a** 24 h mortality; **b** 48 h mortality; **c** 72 h mortality; **d** Percent of mosquitoes that pupated up to 9 days after introduction into the bioassay cages

Larval mortality at 24 h (Fig. 3a and Additional file 1: Table S1) was > 85% for all treatments except the untreated control (0.7%) and the Natular T30 formulation (4.7%) immediately after application of larvicide (week 0). However, mortality at 24 h declined rapidly for all treatments the following week and was < 25% for all treatments except for the Natular T30 formulation, for which mortality at 24 h increased to 54.0% at week 1 and was > 90% for weeks 2 through 4. Monitoring of the Natular EC formulation was planned to continue weekly but stopped after week 1 because mortality was low even after 48 and 72 h and because mosquito availability was limited. Mortality at 24 h of the Natular XRG was 24.7% at week 1 but rose to 52.0% at week 2 and 59.3% at week 3 before declining to 26.0% at week 4.

Immediately after application of the larvicide (week 0), mortality at 48 h (Fig. 3b) was > 70% for all treatments except for the untreated control. As with the 24-h mortality, mortality at 48 h declined rapidly for the Natular EC formulation to 26.7% at 1 week after larvicide application. Mortality at 48 h declined to 80.0% for the Natular XRG formulation but the decline was much lower compared to Natular EC. Mortality at 48 h for larvae exposed to the Natular XRG formulation gradually declined to 60.0% at 4 weeks post-application. Similar to mortality at 24 h, mortality at 48 h for mosquitoes exposed to the Natular T30 rose to > 90% for weeks 1 to 4 after application. Mortality at 72 h followed a similar trend to that at 48 h (Fig. 3c).

Statistical analysis indicated significant interactions between treatment and week for mortality at 24 h (Additional file 1: Table S2). Conditional estimates indicated significantly higher 24-h mortality at week 0 for the Natular EC formulation compared to the XRG and T30 formulations, while the Natular XRG caused higher mortality than the T30 formulation. By week 1, all the larvicide formulations had significantly higher mortality compared to the untreated controls and the Natular T30 formulation had significantly higher mortality compared to the Natular EC formulation. No other pairwise comparisons were significantly different. For weeks 2 through 4, 24-h mortality for mosquitoes in the Natular T30 treatment was significantly higher than the Natular XRG treatment. There was significantly higher 24-h mortality with both formulations compared to the untreated controls (Additional file 1: Table S3). Mortality at 48 and 72 h reached 100% for several treatments and, therefore, the models for these outcomes did not converge.

The proportions of immatures that pupated after being introduced into the bioassay cages are provided in Fig. 3d and Additional file 1: Table S1. At least 80% of control larvae reached pupation during each week except during week 0 when 71.3% of the control larvae reached pupation. Less than 10% of larvae reached pupation with the exceptions of the Natular EC formulation during week 1 and the Natular T30 formulation in week 0 where 14.0% and 18.0% of larvae reached pupation, respectively.

**Fig. 4:**
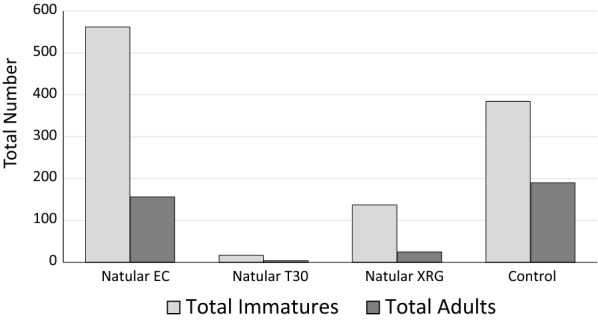
Total number of wild late stage (4th instar and pupae) immatures and adult mosquitoes obtained from habitats outside the bioassay cages from day 11 to day 16 after treatment

The total numbers of 4th instar larvae and pupae along with the total numbers of adults that emerged from outside the bioassay cages are presented in Fig. 4. Over 6 days, 17 and 137 wild 4th instar larvae and pupae were collected in the Natular T30 and Natular XRG treatments, respectively. In contrast, 562 larvae were collected in the Natular EC treatments, and 384 were collected in the control habitats. Only 4 adult *Anopheles* mosquitoes emerged from the Natular T30 treatments and 25 from the Natular XRG treatments, while 156 emerged from the Natular EC treatments and 190 from the control habitats.

### Larval habitat occupancy and larval densities

From April 2011 to September 2012, a total of 1,279 habitats were sampled from a 500 × 500 m grid in the treatment village while 1,037 were identified in a similar grid in the comparison village. The number of habitats sampled at each follow up ranged from 1 to 40 in the control village and from 1 to 136 in the intervention village. At least one larva was observed in 38.3% of habitats in the untreated village, compared to just 3.8% of habitats in the treated village (OR = 0.06, 95% CI = 0.06–0.07, χ^2^ = 2594.2, p < 0.001). Larval densities were lower in the intervention village compared to the control village whether measured as mean per dip, mean per habitat, or mean per m^2^. This trend was similar for early instars, late instars or all instars (Table 1). By negative binomial regression, the number of larvae per habitat was significantly lower in the intervention village for early instar larvae (RR = 0.08, 95%CI = 0.07–0.09, χ^2^ = 1770.1, p < 0.001), late instar larvae (RR = 0.03, 95%CI = 0.03–0.04, χ^2^ = 2019.1, p < 0.001), and total larvae (RR = 0.06, 95%CI = 0.05–0.07, χ^2^ = 3517.5, p < 0.001) (Table 2).

**Table 1:** Summary statistics for evaluation of Spinosad application on habitat occupancy and immature density as measured every 2 weeks in a 500 × 500 m grid within each village

Parameter	Measure	Control	Intervention
Number of habitats	Total habitats	1,037	1,279
Area	Mean area	7.4 (6.7–8.0)	10.5 (8.9–12.2)
	Median area	5.0 (3–8)	6.0 (4–12)
Habitat occupancy	Percent with immatures	38.3 (37.1–39.4)	3.8 (3.5–4.2)
All instars	Mean per dip	1.0 (0.83–1.17)	0.10 (0.06–0.15)
	Mean per habitat	5.46 (4.65–6.26)	0.59 (0.4–0.78)
	Mean per m^2^	1.64 (0.94–2.34)	0.14 (0.02–0.25)
Early instars	Mean per dip	0.83 (0.74–0.93)	0.04 (0.03–0.05)
	Mean per habitat	3.98 (3.48–4.47)	0.18 (0.13–0.22)
	Mean per m^2^	1.41 (0.94–1.86)	0.04 (0.03–0.05)
Late instars	Mean per dip	1.83 (1.61–2.06)	0.14 (0.09–0.19)
	Mean per habitat	9.43 (8.33–10.5)	0.76 (0.56–0.97)
	Mean per m^2^	3.04 (1.99–4.09)	0.18 (0.06–0.3)

**Table 2:** Results of statistical models for habitat occupancy and the number of early (L1 & L2), late (L3, L4 & Pupae) and all instar larvae as measured every 2 weeks in a 500 × 500 m grid within each village

Parameter	Risk Ratio	LowerCL	UpperCL	χ^2^	P-value
Occupancy*					
Scale	2.72	2.72	2.72		< 0.001
Intercept	0.62	0.59	0.65	379.4	< 0.001
Intervention	0.06	0.06	0.07	2594.2	< 0.001
Control	Ref.	Ref.	Ref.		
*Early instars*					
Dispersion	12,121.4	6634.8	23,078.3		< 0.001
Intercept	0.81	0.75	0.87	30.5	< 0.001
Intervention	0.08	0.07	0.09	1770.1	< 0.001
Control	Ref.	Ref.	Ref.		
*Late instars*					
Dispersion	141.22	98.49	208.28		< 0.001
Intercept	0.59	0.55	0.62	299.2	< 0.001
Intervention	0.03	0.03	0.04	2019.1	< 0.001
Control	Ref.	Ref.	Ref.		
*All instars*					
Dispersion	152.25	116.67	201.67		< 0.001
Intercept	1.39	1.32	1.47	134.6	< 0.001
Intervention	0.06	0.05	0.07	3517.5	< 0.001
Control	Ref.	Ref.	Ref.		

Habitat occupancy was compared using logistic regression while the number of larvae per dip was compared using negative binomial regression

^*^The outcome for habitat occupancy is an odds ratio rather than a risk ratio

The trends were similar in the subset of 30 habitats monitored every 4–5 days as for the monitoring of all habitats within the 500 × 500 m grid though the effect was not as strong as that observed in the bi-weekly monitoring. Habitat occupancy in the control village was 56.3% compared to 16.4% in the treated village (OR = 0.15, 95%CI = 0.13–0.17, χ^2^ = 810.4, p < 0.001). The number of larvae per habitat was also significantly lower in the intervention village for early instars (RR = 0.23, 95%CI = 0.20–0.27, χ^2^ = 337.9, p < 0.001), late instars (RR = 0.16, 95%CI = 0.14–0.19, χ^2^ = 579.0, p < 0.001), and total larvae (RR = 0.21, 95%CI = 0.18–0.24, χ^2^ = 455.7, p < 0.001) (Additional file 1: Tables S4 and S5).

### Adult mosquito densities

The mean numbers of mosquitoes collected by PSC are presented in Table 3 by intervention versus control village and by intervention or post-intervention periods. Numbers were generally low for *An. gambiae* and *An. funestus* (< 1 mosquito per house). The number of *Anopheles* mosquitoes collected in the intervention village was lower than the number collected in the control village for both the intervention and post-intervention periods. However, the differences in the number of mosquitoes collected in the intervention *versus* the control village was greater during the intervention period compared to the post-intervention period when larviciding had ceased.

**Table 3:** Summary of adult densities in the control and intervention zones by intervention and post-intervention periods

	Period	Control	Intervention
Number of Collections	Intervention	N = 472	N = 519
	Post-intervention	N = 465	N = 402
Total *Anopheles*	Intervention	1.00 (0.75–1.24)	0.33 (0.26–0.40)
	Post-intervention	0.71 (0.55–0.87)	0.50 (0.28–0.71)
*An. gambiae*	Intervention	0.54 (0.40–0.68)	0.21 (0.16–0.27)
	Post-intervention	0.40 (0.30–0.50)	0.31 (0.17–0.44)
*An. funestus*	Intervention	0.46 (0.31–0.61)	0.12 (0.08–0.16)
	Post-intervention	0.31 (0.20–0.42)	0.19 (0.08–0.30)
Culicines	Intervention	2.35 (1.65–3.04)	0.88 (0.64–1.11)
	Post-intervention	1.54 (1.19–1.89)	0.88 (0.61–1.15)

Results of statistical models that included an interaction term along with conditional estimates to determine the effect of treatment allocation during the intervention and post-intervention periods are shown in Table 4. For all *Anopheles* mosquitoes, the interaction term was statistically significant and conditional regression indicated a significant difference between the villages during the intervention period with approximately 65% fewer mosquitoes in the intervention village compared to the control village (RR = 0.35, 95%CI = 0.23–0.55, Z = − 4.64, p < 0.001). In comparison, there were an estimated 31% fewer *Anopheles* in the intervention village compared to the control village during the post-intervention period, when larviciding was stopped but the difference was not statistically significant (RR = 0.69, 95%CI = 0.43–1.11, Z = − 1.54, p = 0.123).

**Table 4:** Results of a negative binomial regression model for all adult *Anopheles* mosquitoes as measured by pyrethrum spray catches

Parameter	Level	Estimate	LowerCL	UpperCL	Z-value	P-value
Intercept		0.78	0.59	1.04	− 1.71	0.087
Treatment	Intervention	0.69	0.43	1.11	− 1.54	0.123
Treatment	Control	Ref.	Ref.	Ref.		
Period	Intervention	1.45	0.85	2.48	1.37	0.171
Period	Post-intervention	Ref.	Ref.	Ref.		
Treatment*Period	Intervention*Intervention	0.51	0.28	0.94	− 2.17	0.030
Treatment*Period	Intervention*Post-intervention	Ref.	Ref.	Ref.	Ref.	Ref.
Treatment*Period	Control*Intervention	Ref.	Ref.	Ref.	Ref.	Ref.
Treatment*Period	Control*Post-intervention	Ref.	Ref.	Ref.		
NetUse	All under nets	0.79	0.51	1.24	− 1.01	0.311
NetUse	Some under nets	1.24	0.69	2.23	0.73	0.466
NetUse	No one in house	Ref.	Ref.	Ref.		
Eaves	Closed on all sides	0.07	0.01	0.38	− 3.08	0.002
Eaves	Closed on 1–3 sides	0.88	0.17	4.69	− 0.15	0.881
Eaves	Open	Ref.	Ref.	Ref.		
*Conditional effects based on interaction term*						
Treatment conditional on period	Intervention period	0.35	0.23	0.55	− 4.64	< 0.001
Treatment conditional on period	Post-intervention period	0.69	0.43	1.11	− 1.54	0.123

In a further statistical analysis, conditional estimates were made to compare differences over time (intervention versus post-intervention), conditional on the treatment arms (intervention versus control). When comparing intervention and post-intervention time periods conditional on treatment, the number of *Anopheles* collected in houses was lower in the intervention village during the intervention period compared to the post-intervention period (RR = 0.74, 95%CI = 0.48–1.16, Z = − 1.31, p = 0.192) while the number of mosquitoes collected in the control village was higher during the intervention period compared to the post-intervention period (RR = 1.45, 95%CI = 0.85–2.48, Z = 1.37, p = 0.171).

The trend was similar for both *An. gambiae s.l.* (Additional file 1: Table S6) and *An. funestus* (Additional file 1: Table S7). For *An. gambiae s.l*., the interaction term between treatment and period was not statistically significant although in the conditional comparisons, there were 59% fewer mosquitoes in the intervention village during the intervention period and the difference was statistically significant (RR = 0.41, 95%CI = 0.27–0.63, Z = − 4.045, p < 0.001). For the post-intervention period, there were 23% fewer *An. gambiae s.l*. in the intervention village than in the control but the difference was not statistically significant (RR = 0.77, 95%CI = 0.48–1.25, Z = − 1.063, p = 0.288). The trend was strongest in *An. funestus* where the interaction between village and period was statistically significant and there were an estimated 74% fewer *An. funestus* in the treated village during the intervention period (RR = 0.26, 95%CI = 0.14–0.49, Z = − 4.197, p < 0.001). During the post-intervention period, there were 41% fewer *An. funestus* in the treated village, but the difference was not statistically significant (RR = 0.59, 95%CI = 0.30–1.13, Z = − 1.585, p = 0.113).

In the models that included net use and presence of fully closed eaves as covariates, net use was not associated with reduced numbers of *An. gambiae*, *An. funestus* or total *Anopheles* spp. (p > 0.05 for all comparisons). However, the total number of *Anopheles* (RR = 0.07, 95%CI = 0.01–0.38, Z = − 3.08, p = 0.002) and the total number of *An. gambiae s.l.* (RR = 0.11, 95%CI = 0.02–0.69, Z = − 2.359, p = 0.018) were significantly lower in houses with fully closed eaves. The model for *An. funestus* did not converge with the eaves variable included, as there were no *An. funestus* collected from houses with closed eaves.

## Discussion

This study demonstrated the long-term effectiveness of two different spinosad formulations: Natular XRG and Natular T30—under semi-natural conditions. The application of these two formulations in a pilot study in one village in western Kenya resulted in significant reductions in larval occupancy, larval densities and adult densities. Mortality of mosquitoes in the Natular XRG treatment measured after 72 h was > 80% for up to three weeks while mortality of mosquitoes in the Natular T30 treatment was 100% through four weeks post application. Mortality was initially relatively low in the Natular T30 treatment, presumably due to the slow release of the active ingredient, which eventually reached a saturation point. Similar observations were made with *Culex quinquefaciatus* where after several days of sublethal effects, the Natular T30 formulation provided 100% efficacy for up to 84 days [28]. In contrast, the Natular EC formulation caused high mortality immediately after application but the mortality rapidly declined within a week. Although mortality was < 100% even after 72 h in most measurements, the assay likely underestimates mortality in natural settings as the exposure only included 3rd instar larvae and was for only three days. Longer exposures to each formulation beginning as early instar larvae and continuing through the development to the adult stage would likely result in much higher mortality. This is supported by the data on adult emergence as > 85% of larvae that survived the three-day exposure and were subsequently held for up to 9 days after introduction into the bioassay cages failed to emerge. However, the semi-field assay provides a simple, standardized approach to estimate the relative effectiveness of different larvicide formulations.

Larviciding in the field resulted in significant reductions in larval occupancy and larval densities. The application of the two longer lasting formulations of spinosad to a single village in western Kenya over the course of 18 months resulted in significantly lower numbers of indoor resting *Anopheles* mosquitoes compared to a neighboring untreated village. After larviciding was stopped, mosquito numbers remained lower in the former intervention village compared to the control villages although the differences were not statistically significant. Furthermore, though not statistically significant, the number of adult mosquitoes captured in the control village during the post-intervention period relative to the intervention period declined while the number of adult mosquitoes captured in the intervention village increased after larviciding was stopped. This suggests that mosquito densities in the intervention village were substantially suppressed and after release from the intervention, densities increased despite a natural decline that was occurring in the neighboring village. Furthermore, the field application of larviciding was done in a single village with an estimated area of 4.6 km^2^. No buffer zone was included in the study and therefore, the effect of larviciding may have been diluted due to immigration of adult mosquitoes from neighboring villages.

The effects of spinosad on larval and adult mosquitoes observed in this study falls within the range reported from other studies of larval source management in sub-Saharan Africa. One of the first entomological trials of larviciding was also conducted in western Kenya where larval densities were reduced by 95% and adult densities by 92% [29]. A trial in Eritrea also recorded significant reductions in larval and adult *An. arabiensis* [30]. Subsequent studies in the highlands of western Kenya and in urban Dar es Salaam demonstrated impacts on the density of larval and adult *Anopheles* mosquitoes as well as on the incidence of new infections [31] and parasite prevalence [32, 33]. However, no effect of larviciding was observed on adult density, clinical malaria or anemia in a trial in The Gambia despite an 88% reduction in larval densities [34]. It was postulated that the large habitats that had areas that were inaccessible for hand dispersal of larvicide combined with long distance movement of mosquitoes may have reduced the efficacy of larval source management in that setting.

The previous studies that demonstrated a substantial impact on adult mosquitoes and/or malaria outcomes in people were generally done in semi-arid [30], highland [31], or urban settings [32, 33]. The lack of impact on adult densities or clinical malaria in The Gambia highlights the challenges of implementing larval source management throughout all of sub-Saharan Africa. Results from the current study also suggest larval control programmes are most likely to be successful in areas where habitats are “few, fixed and findable” as the impact was greater on adult densities of *An. funestus*, which is generally found in larger, permanent and semi-permanent habitats. However, there was still a substantial effect on adult *An. gambiae s.l*., and a previous trial in lowland western Kenya resulted in even greater impacts on adult populations that were predominantly *An. gambiae s.l*. [29]. More recently, a trial in rural Burkina Faso demonstrated a substantial reduction in adult densities of *Anopheles*, with *An. gambiae s.l*. as the predominant species [35]. Together, these studies suggest that the WHO guidance for the implementation of larval source management only in areas where the habitats are “few, fixed and findable” may need to be revised. At the very least, the guidance could be interpreted more broadly than it has been to date.

In addition to questions about the effectiveness of larval source management in diverse ecological settings, a key concern raised in WHO guidance on larviciding is the feasibility of implementing this strategy. To date, trials of larviciding have relied on weekly application by ground-based staff who must regularly survey their targeted areas to find and treat potential larval habitats. Furthermore, sustaining a larval source management programme would likely require substantial supervision to ensure continued high-quality coverage of these targeted areas. These factors would suggest that larval source management would only be cost-effective in areas with low density of habitats or a high density of people. However, labour costs in much of sub-Saharan Africa are low and analyses of previous studies suggest that the cost-effectiveness of larviciding may be similar to or even greater than ITNs or IRS [36, 37]. New technologies may increase the feasibility of larviciding including alternative insecticides with longer durations of effectiveness such as the formulations of spinosad presented in the current study. Long-lasting formulations of *Bti* are also available [38], as are formulations of pyriproxyfen [39] which was used to successfully reduce malaria in Sri Lanka [40]. Although these longer lasting formulations may not reduce the need for frequent surveys of habitats in all settings, particularly where frequent rains may dilute the larvicide concentration in treated habitats and potentially result in the rapid formation of new untreated habitats [41], their incorporation into larviciding programmes may provide additional operational flexibility. Finally, other technical advances such as the use of drones and/or remote sensing may improve the accuracy of detecting larval habitats and potentially the targeting of these habitats as their range and payload capacity increase. Model programmes for implementing larviciding exist and their experiences have been well documented [42, 43]. However, incorporating newer technologies will require investment in human capital to design, implement and monitor programmes that are adapted to specific ecological settings [44].

This study had several limitations. The conditions of the semi-natural experiment may not have been representative of natural larval habitats, which often experience extreme fluctuations in water level. While some rainfall occurred during the experiment, the water levels were stable. In nature, extreme flooding may dilute the dose of larvicide while drying of habitats could inactivate the active ingredient. The experiment used a laboratory strain of *An. gambiae,* which may have been more susceptible to the insecticides tested. However, few wild mosquitoes were collected from outside the bioassay cages treated with Natular XRG and Natular T30 suggesting that little or no natural resistance to spinosad exists in wild mosquito populations. For the field study, only two villages—one intervention and one control—were included in the study, and the lack of replicate clusters increases the likelihood of spurious results. Furthermore, baseline data were not collected before implementing larviciding. However, monitoring of adult mosquito populations continued after larviciding was ceased and during the post-intervention period, adult mosquito densities increased in the intervention village but decreased in the control village suggesting a strong effect on mosquito populations during the intervention period. Although it would have been preferable to conduct the baseline before the pilot was implemented, the post-intervention follow up does provide an indication of the comparability of the two villages. The only potential drawback to this approach is the potential for residual contamination of larviciding in the treated village. However, the increasing densities of mosquitoes in the intervention village, combined with decreasing densities in the control village in the post-intervention period suggest this was not the case. While the monitoring of adult populations continued after larviciding ceased, the monitoring of larval mosquito densities did not; and therefore, it was not possible to assess whether differences in mosquito densities were due to the treatment or whether there were innate ecological differences between the two villages. Finally, no buffer zone was included in the study design and the impact on adult densities may have been underestimated due to immigration of mosquitoes from neighbouring, untreated villages.

## Conclusions

This study demonstrated the efficacy of two extended release formulations of spinosad in semi-natural habitats and piloted their effectiveness against wild larval and adult *Anopheles* mosquitoes in a village in western Kenya. These larvicide formulations may be an effective tool for programmes implementing larval source management in sub-Saharan Africa and may be particularly effective in areas with larger, permanent or semi-permanent habitats. However, larger, randomized controlled trials of spinosad and/or monitoring of larval source management programs that implement spinosad are necessary to confirm the efficacy and effectiveness of this larvicide for reducing adult mosquito densities and malaria transmission.

## Supplementary information

**Additional file 1: Table S1.** Summary of mortality and overall pupation by week and larvicide treatment. Percent (%) mortality/pupation is presented by week and treatment with lower and upper 95% confidence limits in parentheses. **Table S2.** Results of logistic regression model for 24-hour mortality in the semi-field experiment. **Table S3.** Conditional pairwise mortality comparisons for 24-hour mortality observed in the semi-field experiment. **Table S4.** Summary statistics for evaluation of spinosad application on habitat occupancy and larval density as measured every 4-5 days in 30 sentinel habitats in each village. **Table S5.** Results of statistical models for habitat occupancy and the number of early (L1 & L2), late (L3, L4 & Pupae) and all instar larvae as measured every 4-5 days in 30 sentinel habitats in each village. **Table S6.** Results of a negative binomial regression model for adult *Anopheles gambiae* s.l. mosquitoes as measured by pyrethrum spray catches. **Table S7.** Results of a negative binomial regression model for adult *Anopheles funestus* mosquitoes as measured by pyrethrum spray catches.

## Data Availability

The datasets used and/or analysed during the current study are available from the corresponding author upon reasonable request.

## Abbreviations

*Bti*: *Bacillus thuringiensis* Var. *israelensis*
ITN: Insecticide treated net
IRS: Indoor residual spraying
WHO: World Health Organization
*Bs*: *Bacillus sphaericus*
T30: Dispersible tablet formulation
XRG: Extended release granular formulation
EC: Emulsifiable concentrate formulation
WDG: Water dispersable granular formulation
HDSS: Health and Demographic Surveillance System
KEMRI: Kenya Medical Research Institute
CDC: Centers for Disease Control and Prevention
GIS: Geographic information system
PSC: Pyrethrum spray catch
PDA: Personal digital assistant
RR: Risk ratio
CI: Confidence interval
