# Supplementary material for: Efficacy of extended release formulations of Natular™ (spinosad) against larvae and adults of Anopheles mosquitoes in western Kenya
Source: Malar J. 2020 Nov 26;19:436. doi: 10.1186/s12936-020-03507-y (PMC7691113; doi:10.1186/s12936-020-03507-y)
Supplement: Supplementary file 1 — Additional file 1: Table S1. Summary of mortality and overall pupation by week and larvicide treatment. Percent (%) mortality/pupation is presented by week and treatment with lower and upper 95% confidence limits in parentheses. Table S2. Results of logistic regression model for 24-hour mortality in the semi-field experiment. Table S3. Conditional pairwise mortality comparisons for 24-hour mortality observed in the semi-field experiment. Table S4. Summary statistics for evaluation of spinosad application on habitat occupancy and larval density as measured every 4-5 days in 30 sentinel habitats in each village. Table S5. Results of statistical models for habitat occupancy and the number of early (L1 & L2), late (L3, L4 & Pupae) and all instar larvae as measured every 4-5 days in 30 sentinel habitats in each village. Table S6. Results of a negative binomial regression model for adult Anopheles gambiae s.l. mosquitoes as measured by pyrethrum spray catches. Table S7. Results of a negative binomial regression model for adult Anopheles funestus mosquitoes as measured by pyrethrum spray catches. [file 12936_2020_3507_MOESM1_ESM.docx]

| **Week** | **Treatment** | **Mortality at 24 Hours** | **Mortality at 48 Hours** | **Mortality at 72 Hours** | **Overall**  **Pupation** |
| --- | --- | --- | --- | --- | --- |
| Week 0 | Natular EC | 99.3 (98.1-100) | 100 (100-100) | 100 (100-100) | 0 (0-0) |
| Week 0 | Natular T30 | 4.7 (0.4-8.9) | 74.0 (50.5-97.5) | 78.7 (53.7-100) | 18 (0-39.4) |
| Week 0 | Natular XRG | 85.3 (65-100) | 99.3 (98.1-100) | 99.3 (98.1-100) | 0.7 (0-1.9) |
| Week 0 | Control | 0.7 (0-1.9) | 0.7 (0-1.9) | 0.7 (0-1.9) | 71.3 (59.5-83.2) |
| Week 1 | Natular EC | 14.0 (5.5-22.5) | 26.7 (11.7-41.7) | 46.0 (26.1-65.9) | 14.0 (4.8-23.2) |
| Week 1 | Natular T30 | 54.0 (16.8-91.2) | 93.3 (88.1-98.6) | 98.0 (94.2-100) | 0 (0-0) |
| Week 1 | Natular XRG | 24.7 (6.8-42.5) | 80.0 (70.7-89.3) | 90.0 (84.7-95.3) | 2.0 (0.5-3.5) |
| Week 1 | Control | 0.7 (0-1.9) | 1.3 (0-2.9) | 5.3 (2.8-7.8) | 90.7 (88.3-93) |
| Week 2 | Natular T30 | 94.7 (89.3-100) | 98.7 (96-100) | 100 (100-100) | 0 (0-0) |
| Week 2 | Natular XRG | 52.0 (34.4-69.6) | 78.0 (65.7-90.3) | 84.7 (75.2-94.2) | 3.3 (0.4-6.3) |
| Week 2 | Control | 1.3 (0-4) | 2.7 (0-5.9) | 4.0 (0.1-7.9) | 82.7 (75.6-89.7) |
| Week 3 | Natular T30 | 99.3 (98-100) | 100 (100-100) | 100 (100-100) | 0 (0-0) |
| Week 3 | Natular XRG | 59.3 (36.8-81.9) | 72.0 (53.9-90.1) | 85.3 (73-97.6) | 6.0 (0.7-11.3) |
| Week 3 | Control | 3.3 (0-7.5) | 3.3 (0-7.5) | 4.0 (0-9.3) | 89.3 (81.7-97) |
| Week 4 | Natular T30 | 98.7 (96-100) | 100 (100-100) | 100 (100-100) | 0 (0-0) |
| Week 4 | Natular XRG | 26.0 (13.7-38.3) | 60.0 (44.2-75.8) | 68.0 (56.2-79.8) | 7.3 (1.6-13) |
| Week 4 | Control | 2.7 (0.2-5.1) | 4.7 (1.3-8) | 5.3 (2-8.7) | 81.3 (65.3-97.4) |

**Table S1.** Summary of mortality and overall pupation by week and larvicide treatment. Percent (%) mortality/pupation is presented by week and treatment with lower and upper 95% confidence limits in parentheses.

**Table S2.** Results of logistic regression model for 24-hour mortality in the semi-field experiment.

| **Parameter** | **Odds Ratio** | **LowerCL** | **UpperCL** | **Z** | **P-value** |
| --- | --- | --- | --- | --- | --- |
| Intercept | 0.007 | 0.001 | 0.04 | -5.557 | <0.001 |
| Natular T30 | 7.3 | 1.01 | 52.7 | 1.970 | 0.049 |
| Natular XRG | 866.9 | 85.0 | 8844.9 | 5.709 | <0.001 |
| Natular EC | 22201.0 | 1830.0 | 269335.1 | 7.859 | <0.001 |
| Control | Ref. | Ref. | Ref. |  |  |
| Week 1 | 1.0 | 0.06 | 16.3 | 0.000 | 1.000 |
| Week 2 | 2.0 | 0.12 | 33.1 | 0.490 | 0.624 |
| Week 3 | 5.1 | 0.44 | 59.9 | 1.306 | 0.191 |
| Week 4 | 4.1 | 0.64 | 26.0 | 1.489 | 0.136 |
| Week 0 | Ref. | Ref. | Ref. |  |  |
| Natular T30 x Week 1 | 24.0 | 0.8 | 741.3 | 1.815 | 0.070 |
| Natular T30 x Week 2 | 180.1 | 8.3 | 3918.4 | 3.305 | 0.001 |
| Natular T30 x Week 3 | 592.4 | 16.2 | 21657.4 | 3.477 | 0.001 |
| Natular T30 x Week 4 | 370.3 | 26.7 | 5131.5 | 4.410 | <0.001 |
| Natular T30 x Week 0 | Ref. | Ref. | Ref. |  |  |
| Natular XRG x Week 1 | 0.06 | 0.002 | 1.72 | -1.650 | 0.099 |
| Natular XRG x Week 2 | 0.09 | 0.003 | 3.03 | -1.337 | 0.181 |
| Natular XRG x Week 3 | 0.05 | 0.002 | 1.32 | -1.794 | 0.073 |
| Natular XRG x Week 4 | 0.02 | 0.001 | 0.15 | -3.547 | <0.001 |
| Natular XRG x Week 0 | Ref. | Ref. | Ref. |  |  |
| Natular EC x Week 1 | 0.001 | 0.000 | 0.038 | -3.775 | <0.001 |
| Natular EC x Week 0 | Ref. | Ref. | Ref. |  |  |
| Control x Week 1 | Ref. | Ref. | Ref. |  |  |
| Control x Week 2 | Ref. | Ref. | Ref. |  |  |
| Control x Week 3 | Ref. | Ref. | Ref. |  |  |
| Control x Week 4 | Ref. | Ref. | Ref. |  |  |
| Control x Week 0 | Ref. | Ref. | Ref. |  |  |

**Table S3.** Conditional pairwise mortality comparisons for 24-hour mortality observed in the semi-field experiment.

| **Week** | **Treatment** | **Treatment Mortality** | **Reference** | **Reference Mortality** | **Odds Ratio** | **Lower** | **Upper** | **P-Value** |
| --- | --- | --- | --- | --- | --- | --- | --- | --- |
| 0 | Natular T30 | 4.7 (0.3-9) | Natular XRG | 85.3 (64.6-100) | 0.01 | 0 | 0.05 | <0.001 |
| 0 | Natular T30 | 4.7 (0.3-9) | Natular EC | 99.3 (98.1-100) | <0.01 | <0.01 | <0.01 | <0.001 |
| 0 | Natular T30 | 4.7 (0.3-9) | Control | 0.7 (0-1.9) | 7.29 | 1.01 | 52.7 | 0.049 |
| 0 | Natular XRG | 85.3 (64.6-100) | Natular EC | 99.3 (98.1-100) | 0.04 | 0 | 0.4 | 0.006 |
| 0 | Natular XRG | 85.3 (64.6-100) | Control | 0.7 (0-1.9) | 866.9 | 85.0 | 8844.9 | <0.001 |
| 0 | Natular EC | 99.3 (98.1-100) | Control | 0.7 (0-1.9) | 22201 | 1830 | 269335 | <0.001 |
| 1 | Natular T30 | 54.0 (16-92) | Natular XRG | 24.7 (6.5-42.8) | 3.59 | 0.68 | 18.8 | 0.131 |
| 1 | Natular T30 | 54.0 (16-92) | Natular EC | 14.0 (5.3-22.7) | 7.21 | 1.54 | 33.7 | 0.012 |
| 1 | Natular T30 | 54.0 (16-92) | Control | 0.7(0-1.9) | 174.9 | 18.5 | 1658.3 | <0.001 |
| 1 | Natular XRG | 24.7 (6.5-42.8) | Natular EC | 14.0 (5.3-22.7) | 2.01 | 0.66 | 6.1 | 0.217 |
| 1 | Natular XRG | 24.7(6.5-42.8) | Control | 0.7 (0-1.9) | 48.8 | 6.75 | 352.6 | <0.001 |
| 1 | Natular EC | 14.0 (5.3-22.7) | Control | 0.7 (0-1.9) | 24.3 | 3.69 | 159.5 | 0.001 |
| 2 | Natular T30 | 94.7 (89.3-100) | Natular XRG | 52.0 (34.4-69.6) | 16.4 | 5.31 | 50.6 | <0.001 |
| 2 | Natular T30 | 94.7 (89.3-100) | Control | 1.3 (0-4) | 1313.5 | 176.0 | 9805 | <0.001 |
| 2 | Natular XRG | 52.0 (34.4-69.6) | Control | 1.3 (0-4) | 80.2 | 12.2 | 526.7 | <0.001 |
| 3 | Natular T30 | 99.3 (98-100) | Natular XRG | 59.3 (36.8-81.9) | 102.1 | 14.6 | 716.8 | <0.001 |
| 3 | Natular T30 | 99.3 (98-100) | Control | 3.3 (0-7.5) | 4321 | 526.6 | 35454 | <0.001 |
| 3 | Natular XRG | 59.3 (36.8-81.9) | Control | 3.3 (0-7.5) | 42.3 | 10.29 | 173.9 | <0.001 |
| 4 | Natular T30 | 98.7 (96-100) | Natular XRG | 26.0 (13.7-38.3) | 210.6 | 32.7 | 1358.2 | <0.001 |
| 4 | Natular T30 | 98.7 (96-100) | Control | 2.7 (0.2-5.1) | 2701 | 378.1 | 19296 | <0.001 |
| 4 | Natular XRG | 26.0 (13.7-38.3) | Control | 2.7 (0.2-5.1) | 12.8 | 4.66 | 35.3 | <0.001 |

**Table S4.** Summary statistics for evaluation of spinosad application on habitat occupancy and larval density as measured every 4-5 days in 30 sentinel habitats in each village.

| **Parameter** | **Measure** | **Control** | **Intervention** |
| --- | --- | --- | --- |
| Number of habitats | Total habitats | 2,584 | 2,601 |
| Area | Mean area | 28.1 (25.3-30.8) | 28.4 (26.3-30.4) |
|  | Median area | 9 (4-16) | 16 (8-33) |
| Habitat Occupancy | Percent with immatures | 56.3 (54.4-58.2) | 16.4 (15-17.8) |
| All instars | Mean per dip | 0.45 (0.41-0.5) | 0.09 (0.07-0.11) |
|  | Mean per habitat | 4.56 (4.08-5.04) | 1.07 (0.86-1.27) |
|  | Mean per m^2^ | 0.74 (0.17-1.32) | 0.08 (0.06-0.1) |
| Early instars | Mean per dip | 0.27 (0.24-0.3) | 0.04 (0.03-0.05) |
|  | Mean per habitat | 2.35 (2.12-2.57) | 0.38 (0.32-0.45) |
|  | Mean per m^2^ | 0.29 (0.24-0.34) | 0.04 (0.03-0.05) |
| Late instars | Mean per dip | 0.72 (0.65-0.79) | 0.13 (0.1-0.15) |
|  | Mean per habitat | 6.91 (6.25-7.56) | 1.45 (1.21-1.69) |
|  | Mean per m^2^ | 1.03 (0.46-1.61) | 0.12 (0.1-0.15) |

**Table S5.** Results of statistical models for habitat occupancy and the number of early (L1 & L2), late (L3, L4 & Pupae) and all instar larvae as measured every 4-5 days in 30 sentinel habitats in each village.

| **Parameter** | **Risk Ratio** | **LowerCL** | **UpperCL** | **χ^2^** | **P-value** |
| --- | --- | --- | --- | --- | --- |
| *Occupancy** | |  |  |  |  |
| Scale | 2.72 | 2.72 | 2.72 |  | <0.001 |
| Intercept | 1.29 | 1.19 | 1.39 | 40.9 | <0.001 |
| Intervention | 0.15 | 0.13 | 0.17 | 810.4 | <0.001 |
| Control | Ref. | Ref. | Ref. |  |  |
| *Early Instars* |  |  |  |  |  |
| Dispersion | 1829.8 | 1164.8 | 2958.9 |  | <0.001 |
| Intercept | 4.56 | 4.1 | 5.08 | 769.3 | <0.001 |
| Intervention | 0.23 | 0.20 | 0.27 | 337.9 | <0.001 |
| Control | Ref. | Ref. | Ref. |  |  |
| *Late Instars* |  |  |  |  |  |
| Dispersion | 348.5 | 230.1 | 544.6 |  | <0.001 |
| Intercept | 2.35 | 2.13 | 2.59 | 299.8 | <0.001 |
| Intervention | 0.16 | 0.14 | 0.19 | 579.0 | <0.001 |
| Control | Ref. | Ref. | Ref. |  |  |
| *All Instars* |  |  |  |  |  |
| Dispersion | 675.8 | 470.5 | 991.6 |  | <0.001 |
| Intercept | 6.91 | 6.25 | 7.63 | 1448.9 | <0.001 |
| Intervention | 0.21 | 0.18 | 0.24 | 455.7 | <0.001 |
| Control | Ref. | Ref. | Ref. |  |  |

Habitat occupancy was compared using logistic regression while the number of larvae per dip was compared using negative binomial regression.

*The outcome for habitat occupancy is an odds ratio rather than a risk ratio.

**Table S6.** Results of a negative binomial regression model for adult *Anopheles gambiae* s.l. mosquitoes as measured by pyrethrum spray catches.

| **Parameter** | **Level** | **Risk Ratio** | **LowerCL** | **UpperCL** | **Z-value** | **ProbZ** |  |
| --- | --- | --- | --- | --- | --- | --- | --- |
| Intercept |  | 0.45 | 0.34 | 0.59 | -5.763 | <0.001 |  |
| Treatment | Intervention | 0.77 | 0.48 | 1.25 | -1.063 | 0.288 |  |
| Treatment | Control | Ref | Ref | Ref |  |  |  |
| Period | Intervention | 1.45 | 0.86 | 2.43 | 1.401 | 0.161 |  |
| Period | Post-intervention | Ref | Ref | Ref |  |  |  |
| Treatment*Period | Intervention*Intervention | 0.54 | 0.29 | 1 | -1.946 | 0.052 |  |
| Treatment*Period | Intervention*Post-intervention | Ref | Ref | Ref |  |  |  |
| Treatment*Period | Control*Intervention | Ref | Ref | Ref |  |  |  |
| Treatment*Period | Control*Post-intervention | Ref | Ref | Ref |  |  |  |
| NetUse | All under nets | 0.76 | 0.49 | 1.17 | -1.242 | 0.214 |  |
| NetUse | Some under nets | 1.07 | 0.58 | 1.96 | 0.216 | 0.829 |  |
| NetUse | No one in house | Ref | Ref | Ref |  |  |  |
| Eaves | Closed on all sides | 0.11 | 0.02 | 0.69 | -2.359 | **0.018** |  |
| Eaves | Closed on 1-3 sides | 0.71 | 0.21 | 2.40 | -0.555 | 0.579 |  |
| Eaves | Open | Ref | Ref | Ref |  |  |  |
| *Conditional effects based on interaction term*: | |  |  |  |  |  |  |
| Treatment conditional on period | Intervention | 0.41 | 0.27 | 0.63 | -4.045 | **<0.001** |  |
| Treatment conditional on period | Post-intervention | 0.77 | 0.48 | 1.25 | -1.063 | 0.288 |  |

**Table S7.** Results of a negative binomial regression model for adult *Anopheles funestus* mosquitoes as measured by pyrethrum spray catches*.*

| **Parameter** | **Level** | **Risk Ratio** | **LowerCL** | **UpperCL** | **Z-value** | **ProbZ** |  |
| --- | --- | --- | --- | --- | --- | --- | --- |
| Intercept |  | 0.32 | 0.2 | 0.52 | -4.721 | <0.001 |  |
| Treatment | Intervention | 0.59 | 0.3 | 1.13 | -1.585 | 0.113 |  |
| Treatment | Control | Ref | Ref | Ref |  |  |  |
| Period | Intervention | 1.5 | 0.74 | 3.02 | 1.124 | 0.261 |  |
| Period | Post-intervention | Ref | Ref | Ref |  |  |  |
| Treatment*Period | Intervention*Intervention | 0.45 | 0.21 | 0.99 | -1.986 | **0.047** |  |
| Treatment*Period | Intervention*Post-intervention | Ref | Ref | Ref |  |  |  |
| Treatment*Period | Control*Intervention | Ref | Ref | Ref |  |  |  |
| Treatment*Period | Control*Post-intervention | Ref | Ref | Ref |  |  |  |
| NetUse | All under nets | 0.83 | 0.46 | 1.51 | -0.605 | 0.545 |  |
| NetUse | Some under nets | 1.59 | 0.69 | 3.67 | 1.081 | 0.280 |  |
| NetUse | No one in house | Ref | Ref | Ref |  |  |  |
| *Conditional effects based on interaction term*: | |  |  |  |  |  |  |
| Treatment conditional on period | Intervention | 0.26 | 0.14 | 0.49 | -4.197 | **<0.001** |  |
| Treatment conditional on period | Post-intervention | 0.59 | 0.3 | 1.13 | -1.585 | 0.113 |  |
